# An epidemiological approach to mass casualty incidents in the Principality of Asturias (Spain)

**DOI:** 10.1186/s13049-016-0211-x

**Published:** 2016-02-24

**Authors:** Rafael Castro Delgado, Cecilia Naves Gómez, Tatiana Cuartas Álvarez, Pedro Arcos González

**Affiliations:** Unit for Research in Emergency and Disaster, Department of Medicine, University of Oviedo, Campus de El Cristo, Oviedo, 33006 Spain; SAMU-Asturias, Oviedo, Spain

**Keywords:** Mass casualty incidents, Emergency medical services, Disaster epidemiology

## Abstract

**Background:**

Mass Casualty Incidents (MCI) have been rarely studied from epidemiological approaches. The objective of this study is to establish the epidemiological profile of MCI in the autonomous region of the Principality of Asturias (Spain) and analyse ambulance deployment and severity of patients.

**Methods:**

This is a population-based prospective study run in 2014. Inclusion criteria for MCI is “every incident with four or more people affected that requires ambulance mobilisation”.

**Results:**

Thirty-nine MCI have been identified in Asturias in 2014. Thirty-one (79 %) were road traffic accidents, three (7.5 %) fires and five (12.8 %) other types. Twenty-one incidents (56.7 %) had four patients, and only three of them (8 %) had seven or more patients. An average of 2.41 ambulances per incident were deployed (standard error = 0.18). Most of the patients per incident were minor injured patients (mean = 4; standard error = 0.2), and 0,26 were severe patients (standard error = 0.08). There was a positive significant correlation (*p* < 0.01) between the total number of patients and the total number of ambulances deployed and between the total number of patients and Advanced Life Support (ALS) ambulances deployed (*p* < 0.001). The total number of non-ALS ambulances was not related with the total number of patients.

**Discussion:**

Population based research in MCI is essential to define MCI profile. Quantitative definition of MCI, adapted to resources, avoid selection bias and present a more accurate profile of MCI. As espected, road traffic accidents are the most frequent MCI in our region. This aspect is essential to plan training and response to MCI. Analysis of total response to MCI shows that for almost an hour, we should plan extra resources for daily emergencies. This data is an important issue to bear in mind when planning MCI response. The fact that most patients are classified as minor injured and more advanced life support units than needed are deployed shows that analysis of resources deployment and patient severity helps us to better plan future MCI response.

**Conclusions:**

Road traffic accidents with minor injured patients are the most frequent MCI in our region. More advanced life support units than needed have been initially deployed, which might compromise response to daily emergencies during an MCI.

## Background

Mass casualty incidents (MCI) are situations in which research represents a real challenge. One of the greatest difficulties in the study of MCI is the absence of a generally accepted definition in which quantitative criteria is to be included. World Health Organization (WHO) defines MCI as “events which generate more patients at one time than locally available resources can manage using routine procedures. They require exceptional emergency arrangements and additional or extraordinary assistance” [1]. The Department of Health’s Strategic National Guidance to the UK National Health Service for Major Incident Emergency Planning defines a major incident (MI) as “any occurrence that presents a serious threat to the health of the community, disruption to the service or causes such a number or type of casualties so as to require special arrangements to be implemented by hospitals, ambulance trusts or primary care organisations” [2]. These two concepts with similar definitions make difficult to standardise research and publications. Besides, we should also take into consideration that the number of patients is not direct linked to the definition of MCI, which appears to be mostly related to overloaded resources.

Trying to improve research in MCI, the Northern Spain Disaster working group, which is made up of medical professionals responsible for MCI and disasters planning response of eight Emergency Medical Services (EMS) from the north of Spain, and the Unit for Research in Emergency and Disaster of University of Oviedo, developed a uniform reporting template for MCI in Spain [3]. Following a Delphi methodology, inclusion criteria is “incidents with more than four patients”, and 54 variables were defined grouped in four dimensions: (i) MCI description (13 variables); (ii) Prehospital response (23 variables); (iii) Rescue and triage (15 variables); and (iv) improvement actions (3 variables). Template is briefly described in Appendix I.

This study aims to present the results of the first year of work of MCI register, and also characterise the epidemiology of MCI that happened in the Principality of Asturias in 2014, focused on epidemiology, description of the incidents and resource mobilisation.

## Methods

### Study setting

The Principality of Asturias is one of the seventeen autonomous communities of Spain. It is located to the north coast of Spain, covering a surface of 10,604 Km^2^ and with a population of 1,062.000 inhabitants in 2014. A public healthcare system (named Health Service of the Principality of Asturias, SESPA) covers 100 % of the population with a well developed network of primary healthcare centres and eight public hospitals. Prehospital emergency care is provided by SAMU-Asturias, which covers all the territory with basic life support (BLS) and advanced life support (ALS) ambulances. ALS are staffed by physician, a qualified nurse and two basic emergency medical technicians (EMT). Emergency call center is also staffed by physicians together with call operators. Rural areas are mainly covered by primary healthcare teams which staff BLS ambulances. Due to the hight concentration of population in the central area of Asturias, 90 % of the total population can be reached by an ALS ambulance in less than 15 min. In total, eight ALS and 21 BLS ambulances cover all the territory. In addition, an Advanced Medical Post (or sometimes called Field Hospital) can be activated, together with extra medical supplies, 24 h daily all year round. Emergency call centre received 293,145 calls in 2013, 104,429 of which required resource mobilisation (ALS, BLS, Primary health care team or other ambulances). Central Asturias area is affected by industrial risk and nine industrial facilities are affected by the European Union legislation regarding the prevention of major accidents involving dangerous substances [4].

### Study design

This is a population-based prospective study of the MCI occurred in Asturias in 2014. Inclusion criteria for defining MCI is “every incident with four or more people affected that requires ambulance mobilisation”. This inclusion criteria has been defined accordingly to the Northern Spain Disaster working group members using a Delphi methology and according to the structure and resources of our Emergency Medical Services. It has helped us to reduce the objective component of MCI definition and to detect every incident that might be defined as MCI in accordance with more accepted qualitative definitions. A non-public web-based form has been developed to enter all MCI that meet the inclusion criteria.

### Data

Data was collected prospectively from EMS call center registry and personal interviews were made to MCI first responders when needed. Only one person was responsible for detecting MCI and data entry. The doctor who receives the call identifies every incident with four or more patients with a specific icon displayed on the call screen. Data is organised accordingly to the four dimensions mentioned above.

A descriptive statistical analysis using absolute and relative frequencies has been done to establish the profile and characteristics of MCIs. The relationship between MCI and resources used has been studied by Correlation analysis. To study temporal trends we used Linear regression with exponential smoothing to improve data fit. All the statistical analysis was made using SPSS™ statistical software.

For this study, we have used the later European Union Standards regarding medical vehicles and their equipment [5]:

- Ambulance type C: mobile intensive care unit. Road ambulance designed and equipped for the transport, advanced treatment and monitoring of patients. They can be called Advanced Life Support (ALS) ambulances.
- Ambulance type B: emergency ambulance. Road ambulance designed and equipped for the transport, basic treatment and monitoring of patients. They can be called Basic Life Support (BLS) ambulances.
- Ambulance type A: patient transport ambulance. Road ambulance designed and equipped for the transport of patients who are not expected to become emergency patients.

Patients were assigned by a prehospital physician to different categories accordingly to their severity. Categories used were minor injured, moderate, severe and death. They were classified following the Prehospital Advanced Triage Model [6], which is based in the Advanced Trauma Life Support protocol. The equivalences to the color classification are: minor injured-green, moderate-yellow, severe-red and death-black.

## Results

Thirty-nine MCI have been identified in 2014 in Asturias. Thirty-one (79 %) were road traffic accidents, three (7.5 %) fires and five (12.8 %) other type, among which we can find three sinking ships, one marihuana intoxication and one wasp sting. No missing data was observed.

**Fig. 1:**
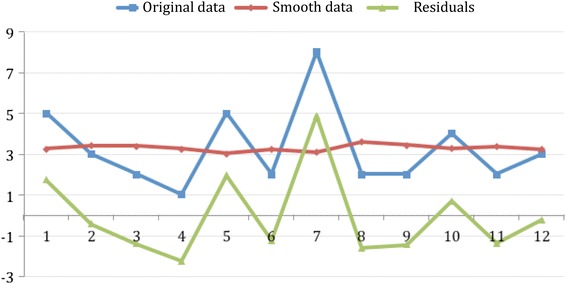
Monthly MCI occurrence and trend in 2014. This figure shows monthly distribution through the year. Although more MCI happens in summer, no statistical significance has been found

A monthly average of 3.2 MCI (standard error = 0.56) have occurred in Asturias throughout 2014. In July, MCI occurrence was almost two and a half times the 2014 monthly average. Regression analysis has shown no significant trend in the evolution of the monthly frequency of MCI throughout the year. Figure 1 shows original, exponential smoothing and residuals series in the MCI.

**Fig. 2:**
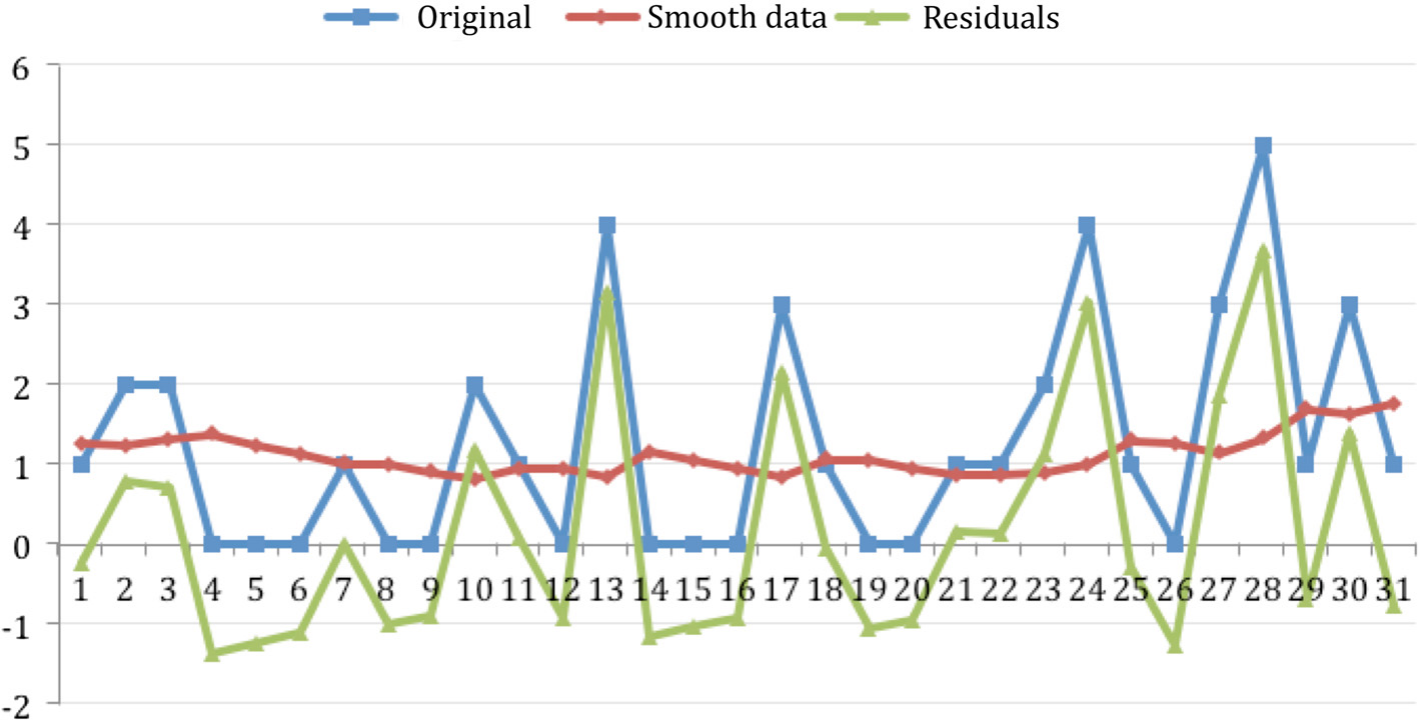
Daily MCI occurrence and trend throughout the month. This figure shows number of MCI each day of the month

**Fig. 3:**
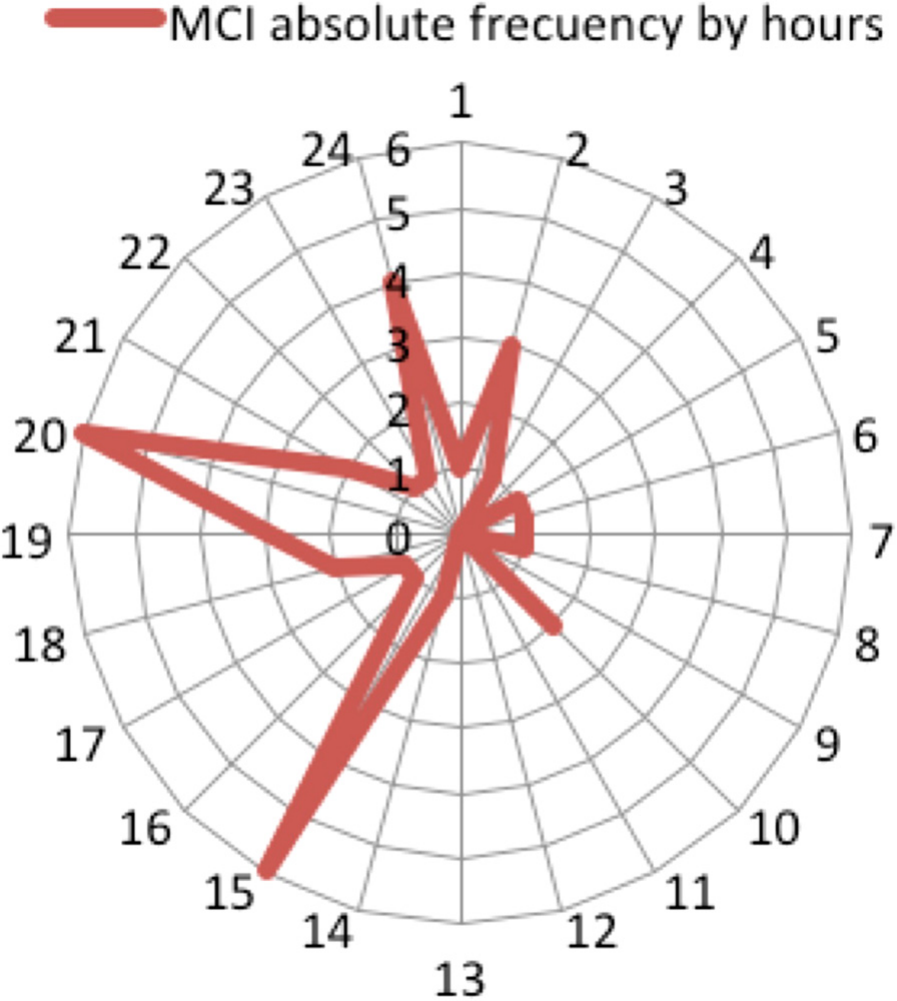
Hourly distribution of MCI (24 h). This figure shows hourly distribution of MCI through the day, with more MCI at noon and late afternoon

Although there was an increase in the frequency of daily MCI throughout the days of the month, this upward trend was not significant. Figure 2 shows original, exponential smoothing and residuals series thoughout the 31 days of the months of 2014. In the distribution of the occurrence of MCI by hours, there is a significant (*p* < 0.05) greater frequency around two day periods: at noon (14:00 to 15:00) and late afternoon (18:00 to 20:00) as shown in Fig. 3.

Chemical toxic substances were involved in three (7.6 %) of the incidents, and no incident was found to have CBRN risk. In 11 (28.2 %) incidents primary health care team was actively involved in the response.

Average duration time of an MCI, defined as the time from first call and back to service of the last ambulance, in Asturias in 2014 was 53 min (standard error = 3.48) with a mean of 53 min (Q1 = 39.5; Q3 = 68).

**Fig. 4:**
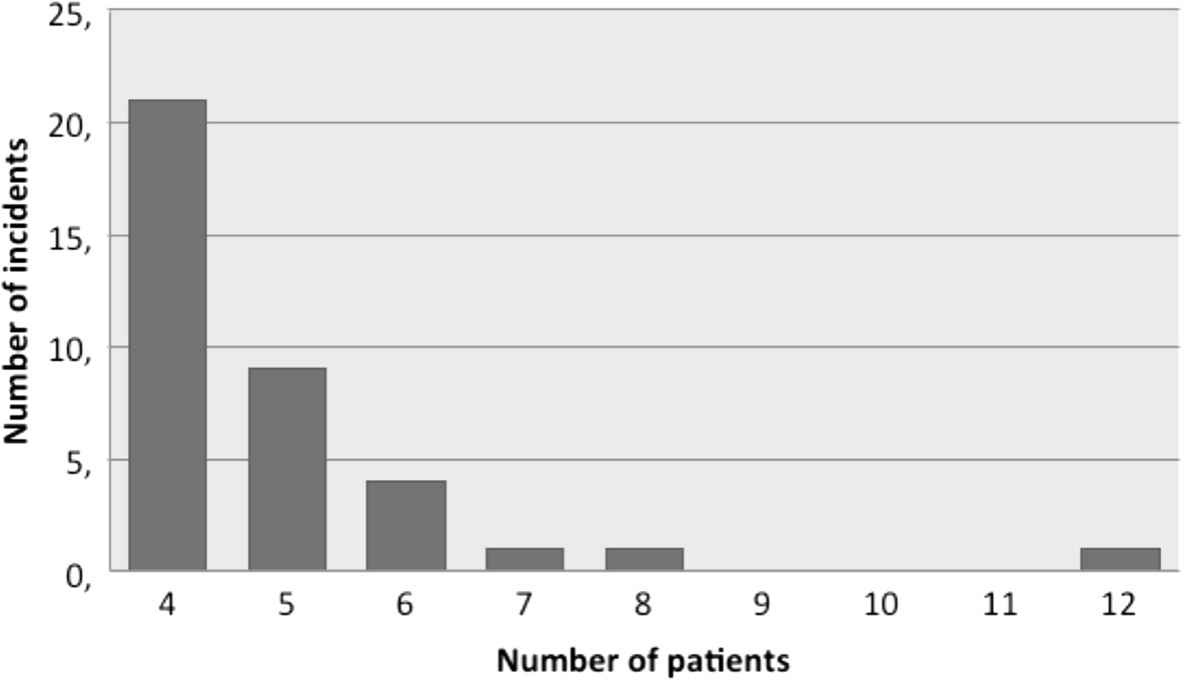
Number of patients per incident. Most of the incidents had only 4 patients, and only a few had more than 7 patients

As shown in Fig. 4 twenty two incidents (56.7 %) had four patients, and only three (8 %) had seven or more patients.

The two incidents with more patients had 12 and eight patients respectively, and both of them were related with sinking ships. The road traffic accident with the most patients had seven patients. Regarding ambulance deployment, an average of 2.4 ambulances were deployed (standard error = 0.18). Most of the patients per incident were minor injured patients (mean = 4; standard error = 0.2), and only 0.26 were severe patients (standard error = 0.08).

The field hospital was deployed only once, due to a sinking ship. Helicopter was deployed in two incidents, both related with sinking ships. A brief summary of main MCI characteristics are shown in table 1.

**Table 1:** Main features of MCI in Asturias in 2014

Incident		Patients		Duration of incident	Ambulances		Injured patients	
Type	n (%)	Patients per incident	Number of incidents (%)	Mean 53 min (SE = 3.48)	Type	Ambulances deployed	Severe	Mean 0.26 (SE = 0.08)
Road traffic accident	31 (79)	4	21 (56)		Type C	Mean 0.66 (SE = 0.11)	Moderate	Mean 0,31 (SE = 0.09)
		5	9 (24)		Type B	Mean 1,23 (SE = 0.12)	Minor injured	Mean 4 (SE = 0,20)
Fire	3 (7.5)	6	4 (10)		Type A	Mean 0.51 (SE = 0.10)	Dead	Mean 0.34 (SE = 0.22)
		7	1 (2.7)		TOTAL	Mean 2.41 (SE = 0.18)	TOTAL	Mean 4.92 (SE = 0.25)
Others	5 (12.8)	8	1 (2.7)					
		12	1 (2.7)					

*SE* standard error

There is a positive significant statistical correlation (*p* < 0.001) between the total number of patients and total number of ambulances deployed. There is also a positive and statistical significant correlation (*p* < 0.0001) between the total number of patients and type C ambulances deployed. No significant relation has been found between the number of ambulances and the type of incident, or between the number of non-ALS ambulances (type B + type A) and non severe patients.

Regarding the number and severity of patients and ambulance mobilisation, there was a positive correlation (*p* < 0.05) between the number of type C ambulances and the number of severe injured patients, and also between the total ambulances deployed and the total number of severe injured patients (*p* < 0.0001). A brief summary of correlations between severity of patients and type of ambulances deployed is represented in table 2.

**Table 2:** Correlations between severity of patients and type of ambulance

	Type C ambulance	Type A + B ambulances	Total ambulances
Severe patients	*r* = 0.39 (*p* = 0.016)	*r* = 0.15 (*p* = 0.35)	*r* = 0.55 (*p* = 0.0004)
Non severe patients	*r* = 0.,15 (*p* = 0.34)	*r* = 0.15 (*p* = 0.35)	*r* = 0.21 (*p* = 0.17)
Total patients	*r* = 0.52 (*p* = 0.0008)	*r* = 0.18 (*p* = 0.28)	*r* = 0.47 (*p* = 0.003)

r, Pearson’s Correlation Coefficient (*p* value)

There was no correlation between duration of the MCI response and the number of patients. The average number of patients in sinking ships MCI was higher (*p* < 0.00001) than in MCI by road traffic accidents. No correlation between the severity of patients and the type of incident, or the type of incident and the ambulances deployed have been found.

## Discussion

### Research in mass casualty incidents

The lack of standardised research of MCI has recently been pointed out by some authors as a gap to improve medical response [7]. Because of the importance of investigation into MCIs, many efforts to centralise registries of MCIs to share information and feedback on lessons learnt in this field have been made worldwide, with not much success [8, 9]. A literature review published in 2013 identifies and describes the content of templates for reporting prehospital major incident medical management, and concludes that “none of them have been tested for feasibility in real-life incidents [10]”. This lack of correlation between recommendations and real-life MCI has also been demonstrated in triage aspects [11] related to MCI.

Recently, new attempts to create a central MI database [12] have made possible the publication of better quality data, which could be a starting point for analysing real MCI/MI data [13]. According to this template, a major incident is defined as “an incident that requires the mobilisation of extraordinary EMS resources and is identified as a major incident in that system”. Another possible definition is “when the number of people involved, the type of incident and the location of the incident require extraordinary rescue efforts”. These are qualitative definitions, which make inclusion criteria more flexible than if they were quantitative definitions. Another problem, as described by the authors, is that there is an important selection bias; it seems that probably only those incidents with the best response or more data available would be recorded. In order to tackle the problem it is necessary to change the approach to MCIs databases. A good starting point would be to transform the definition to a quantitative one, following the disaster definition example from the Center for Research on the Epidemiology of Disasters (CRED). In CRED disaster database, a disaster is defined as any event with: ten or more people reported killed, one hundred or more people reported affected, declaration of a state of emergency or call for international assistance (www.emdat.be). The advantage of that approach is that it is easier to define inclusion criteria, and even different systems could use different inclusion criteria accordingly to their structure and resources so that they could define their own quantitative definition of MCI. Nevertheless, it also has the disadvantage of variability among these different services.

Other authors have published disasters and MCI data from a single database [14]. This approach of mixing Disaster and MCI concepts might lead to a loss of data that could be very interesting for MCI response but not for disasters, or vice versa. Attempts to create a scientific framework for research on disaster and mass casualty incidents have also been recently published [15].

### Mass casualty incident: from research to response

Much research has been made regarding disaster epidemiology and response [16], but little regarding specific MCI epidemiology. However, new studies have been recently published in the United States [17, 18] and Europe [19]. Clinical recommendations are usually based on previous research and case studies, but little research has been made in the field of MCI [20].

Most Emergency Medical Systems have developed their own MCI response protocols, mainly based on internationally accepted ideas and recommendations, but very seldom based on the best evidence practice. For this reason, it is essential to have well established database of MCI that could describes their characteristics, distribution and medical response to adapt protocols to real MCI data and response. To date, this is the first population based research that describes MCI profiles and response with four or more patients.

An interesting discussion that could be related to the limitations of this study is whether a four-patients incident could be considered as an MCI for a developed EMS. We have to consider that if an MCI protocol is activated only with many patients, very seldom resources will apply trained protocols and procedures of MCI response in real incidents. The fact that in our study we analyse all incidents with four or more patients gives us the opportunity to differentiate mild incidents and more severe incidents. This might lead us to improve resource mobilisation in the mild and moderate incidents, which are more frequent. However, we must take into consideration that even in well developed Emergency Medical Services the number of patients in an MCI is determinant to plan the response. This is so because the response to an incident involving five patients is not the same compared to a 400 patients MCI; in this case more complex and multiagency organization is needed. With our inclusion criteria we can decide what type of MCI we want to study, and stablish epidemiological profiles according to their characteristics.

### Mass casualty incident profile

Once this consideration is made regarding inclusion criteria, the fact that the most frequent MCI (with 79 % of the cases) is road traffic accidents shows that EMS response training programmes in MCI should be focused on them. Consequently, most EMS training programmes in MCI response are focused on much less frequent incidents like chemical incidents [21] or terrorism [22]. Despite the high number of industrial facilities located in the area [23], no MCI chemical accident has been reported.

When we analyse yearly distribution, no significant trend can be seen in the evolution. The number of incidents in July has been doubled probably due to an increase of road traffic accidents. There has also been an increase in other type of incidents. The hourly distribution of incidents, with more incidents at noon and late afternoon, is not expected to be only because of an increase of road traffic accidents.

The total response time is calculated from the first emergency call to the last ambulance is available again for any other emergency. The average duration of 53 min shows that after an MCI, EMS could be overloaded for an hour or so. This means that it should be taken into consideration their surge capacity not only to response to the MCI, but also to keep on responding to daily emergencies [24]. The lack of correlation between the total response time and the number of patients could mean that total response time could be influenced by other aspects like location, rescue tasks or risky situations.

Only three incidents had eight or more patients. This shows that if we only study the most severe MCI, we lose many incidents whose data could help us to improve medical response.

### Resources deployment

When we analyse ambulance deployment, as expected, the more patients we have the more ambulances are deployed. However, this is due to a correlation between the number of type C ambulances deployed and the total number of patients (and also with the total number of severe patients), because no relationship between the total number of non-severe patients and the type B + type A ambulances deployed has clearly seen. This might show an overestimation of the incident by the EMS call center, sending initially more ALS than needed. This might compromise response to daily emergencies during an MCI due to an overuse of type C ambulance in MCI. This fact has also been described by El Sayed M. et al. [19]. The lack of correlation between non-ALS ambulances (type B + type A) and the number of patients could be explained because non ALS ambulances can transport more than one patient at a time in case of MCI. The number of ambulances is not related to the type of incident, so initial information regarding type of incident could not be used as a single parameter to decide resources for mobilisation.

The proportion of minor injured patients in our MCI database is similar to previous data published, with higher proportion of minor injured patients [25]. This brings us to the fact that a key aspect of the response could be patient transportation. Another important finding is the participation of primary health care teams in the response. This leads us to recommend the involvement of these teams in the training programmes developed by EMS organizations, so that joint response could be properly trained [26].

The use of helicopter in MCI has seldom been studied [27]. We found that it has only been used in sinking ships incidents, but never in road traffic MCI incidents or fires, most of which were in urban settings.

Other resource whose use has not been well established in urban setting is the use of tent field hospitals. In our case, a field hospital was deployed only once, in an non-urban setting and it was probably due to long rescue times during a sinking ship. This leads us to recommend the establishment of well defined protocols for field hospital deployments in modern EMS; most of them have this resource and its usefulness has not been well defined [28].

With this data we define the pattern on MCI in our region, which can help us to improve training and response by knowing what we are facing. Our data could be useful for European countries with well developed health system and similar social stability. The template used consist of 54 variables which will be analysed more in depth according to the needs and lessons learnt from MCI response. More regions in Spain are starting to use this online template to analyse and compare data. This will be very useful to make research in MCI.

### Strengths and limitations of this study

This is the first population-based study in Europe that describes epidemiological pattern and response to mass casualty incidents in a complete geographical region. Previous studies only include data from a sample of incidents, but not from all incidents occurred in a certain area. Methodology used for data collection avoid missing cases. A very sensitive quantitative definition of mass casualty incident allows us to give a proper approach to their pattern and detect all MCI. We think this quantitative definition is useful for systems and societies with similar features.

Several limitations of this study are worth noting. Firstly, the number of incidents studied, even though MCI are usually a low-frequency phenomenon in developed countries. Then, the fact that the study refers to a specific geographical region. Our quantitative definition of MCIs is adapted to our region and it could not be useful for other regions with different economic, social and cultural context.

## Conclusion

Description of MCI allows us to plan training strategies and response protocols to adapt them to the expected epidemiology and to detect areas to improve in the medical response to MCI. EMS should adapt training programmes and resources to the distribution and frequency of MCI: summer and road traffic accidents in our case. Correlations found between the total number of injured patients, the severity of patients and the ambulance deployment might indicate an overuse of ALS ambulances in MCI. More research should be done focused on MCI profile and response epidemiology to clearly establish needs in that kind of events.

No ethical approval has been needed according to institutional regulations for this case.

This research has been partly funded by the Erasmus Mundus Master in Public Health in Disasters.

## Appendix I

### Summary of template used for reporting mass casualty incidents

1. DESCRIPTION
  - Number of incident
  - Region
  - Province
  - City
  - Time of first call
  - Date
  - Week day
  - Tipe of incident
  - Toxic substances
  - CBRN risk?
  - Causes of MCI
  - Ending time
  - Total duration time
2. PROHOSPITAL RESPONSE
  - Time of arrival of first basic life support unit (BLS)
  - Response time of first BLS
  - Number of BLS
  - Time of arrival of first advanced life support unit (ALS)
  - Response time of first ALS
  - Number of ALS
  - Time of arrival of first medica helicopter (HEMS)
  - Response time of first HEMS
  - Number of HEMS
  - Time of arrival of first Primary Health Care Team (PHCT)
  - Response time of first PHCT
  - Number of PHCT
  - Advanced Medical Post (AMP) deployed?
  - Time of activation of AMP
  - Time of operability of AMP
  - Activation time of AMP
  - Police?
  - Rescue teams?
  - Others?
  - Advanced command and control team?
  - Who is main command and controL person?
  - On scene communications
  - Command and control communications
3. RESCUE AND TRIAGE
  - Who perform rescue?
  - Medical rescue?
  - Triage before rescue?
  - Triage before rescue system used
  - Who perform triage before rescue?
  - Triage after rescue?
  - Triage after rescue system used
  - Who perform triage after rescue?
  - Identification tags used
  - Total number of victims
  - Number of severe victims
  - Number of moderate victims
  - Number of minor injured victims
  - Number of death victims
  - Person who decide evacuation
4. IMPROVEMENT
  - Evaluation meetings
  - Final report
  - Improvement actions
